# Intravenous injection of elemental mercury: A report of two cases

**DOI:** 10.4103/0970-0358.44942

**Published:** 2008

**Authors:** A. Gopalakrishna, T.V. Pavan Kumar

**Affiliations:** Departments of Plastic Surgery, Deccan College of Medical Sciences, Hyderabad, Andhra Pradesh, India; 1 Departments of General Surgery, Deccan College of Medical Sciences, Hyderabad, Andhra Pradesh, India

**Keywords:** Elemental mercury, intravenous injection

## Abstract

Two cases of intravenous injection of elemental mercury are described in this report. One patient succumbed and the other remains asymptomatic two years after the surgical removal of all the injected mercury. Management of intravenous injection of elemental mercury (intended to be an aphrodisiac in these two cases) is discussed here and the need for surgical removal of all accessible mercury has been emphasized.

## INTRODUCTION

Unlike many metals, mercury is a unique element that has no essential biological function. It is liquid at room temperature and has a density of 13.6 g/mL at room temperature. It is used in the manufacture of switches, thermometers, BP apparatus and other instruments, extraction of gold and silver, and has also been known to be used as a preservative and pesticide, as well as in some medications. Exposure to mercury is an occupational hazard in many industries and contamination of air, soil, and water with mercury is a major concern.

Exposure to mercury can occur in many ways: inhalation of its vapor, ingestion of mercury salts, ingestion of elemental mercury (deliberate or accidental), and intravenous injection of elemental mercury. Salts of mercury are more poisonous than elemental mercury and the inhalation of elemental mercury vapors is more hazardous than its ingestion or intravenous injection.[1–5]

Intravenous injection of mercury has been reported for attempted suicide, attempted homicide,[6] and addiction.[7–10] Mercury is used in India by practitioners of alternative medicine such as Ayurveda, siddha and Unani medicine. In these instances, especially Ayurveda, mercury is used in the form of *Bhasma* that is obtained on incinerating mercury along with selected herbs.

The presence of mercury in the environment is due to pollution from power plants and in food, particularly fish, and in dental amalgams.

WHO guidelines maintain that the lowest harmful level of mercury for humans is five parts per million (ppm). The estimation of hair mercury levels is considered to be a valid test for the presence of mercury in the blood; a level < 15 *µ*g/g is considered to be safe.[11]

## CASE REPORTS

**Figure 1:**
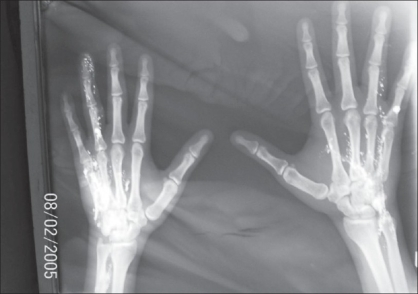
Patient - 1 Radiograph showing mercury deposit

**Figure 2:**
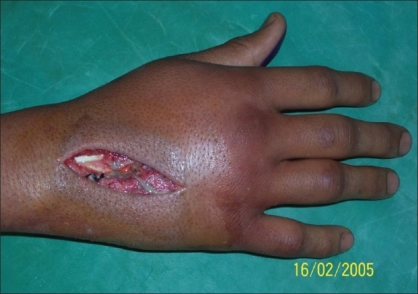
Patient - 2 hand after incision and drainage

**Figure 3:**
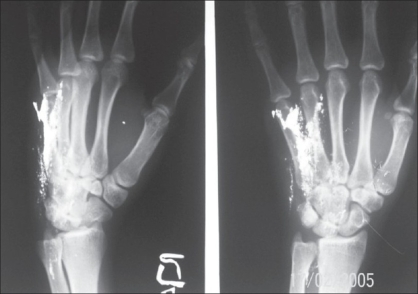
Patient - 2 showing mercury deposits

**Figure 4:**
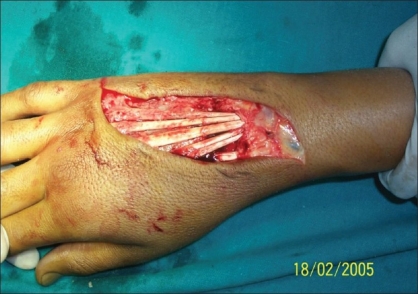
Patient - 2 hand after debridement and removal of mercury

**Case 1:** A 22 year-old trainee in alternative medicine was admitted in our hospital on 8^th^ February 2005 with a history of intravenous injection of elemental mercury in both hands [Figures  1–4]. On examination, the patient was found to have fever, tachycardia, and inflammation of both hands extending up to the forearm. The patient had had an incision and drainage for a supposed abcess in another hospital. Radiography of both hands showed a radio-opaque shadow extending from the tip of the ring finger to the lower third of the radius and ulna on the left hand, and from the PIP joint of the ring finger to the wrist joint of the right hand. The patient left against medical advice before any further investigations or surgical procedures. He was later admitted to a private nursing home and it was reported that he died within 48 h of admission. No further information was available from the relatives except that he had taken many other injections in addition to the mercury, and that he had been doing this for more than two years.

**Figure 5:**
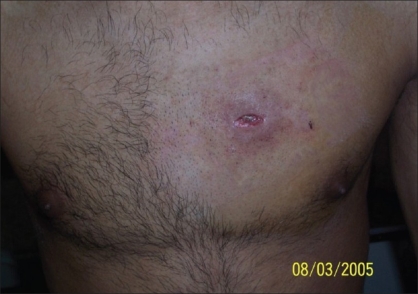
Patient - 2 Inflamed chest wall following incomplete removal of mercury

**Figure 6:**
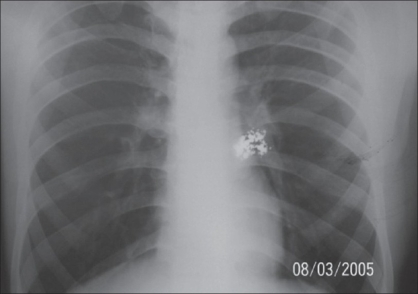
Patient - 2 mercury deposit in anterior chest wall

**Figure 7:**
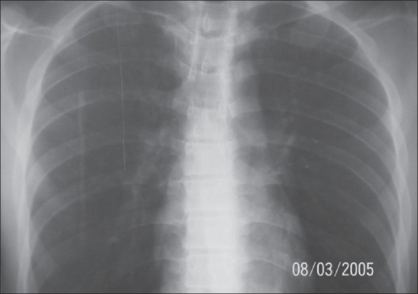
Patient - 2 X-ray chest after granuloma excision

**Figure 8:**
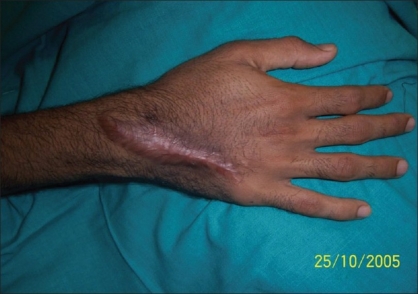
Patient - 2 hand eight months post operative

**Figure 9:**
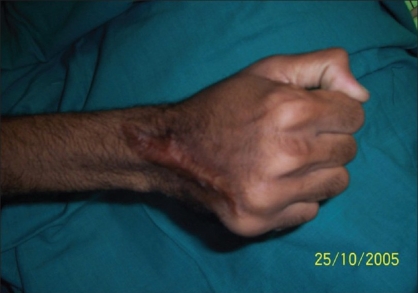
Patient - 2 hand function

**Figure 10:**
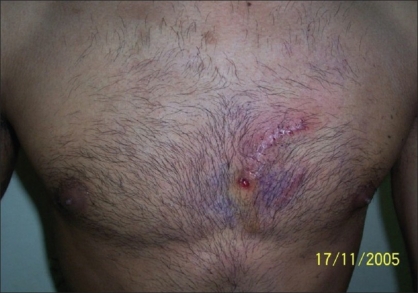
Patient - 2 well healed wound after granuloma excision

**Case 2:** A 20 year-old male was admitted to our hospital on 15^th^ February 2005 with a history of intravenous injection of mercury taken by him and his friend as an aphrodisiac. The patient presented with fever, tachycardia, and swelling of the right hand (which was the primary site of injection) extending up to the forearm [Figures 5–10]. The patient was haemodynamically stable. An attempt had been made for incision and drainage in the emergency room because it was diagnosed as a injection abscess. An X-ray of the hand revealed a radio-opaque shadow overlying the fourth metacarpal, third and fourth intermetacarpal space extending over the carpus, and the wrist joint up to about 1 cm over the distal end of the radius and ulna dorsal to the skeleton. A diagnosis of extravasation of injected mercury was made. After basic investigations, the patient was shifted to the operation theatre where a thorough debridement was performed under general anaesthesia and a large amount of mercury was removed physically from the dorsum of the hand. Some of the mercury could only be removed along with excision of the surrounding soft tissue; the removal of mercury was confirmed by radiography. The wound was left open so that any mercury left behind could find a passage to come out. On the following day, the patient went home against medical advice but came back on the 7^th^ of March with a history of another subcutaneous injection of elemental mercury over the precordium. Once again, an attempt had been made to drain an abscess in another hospital. The patient refused any further surgical procedures although the radiograph showed the deposition of mercury in the subcutaneous plane in the chest wall. The patient turned up in October 2005, with a swelling over the precordium with surrounding inflammation [Figure 10]. This time, he was willing for surgery and the mercury was removed along with surrounding tissue *en masse* and the wound healed uneventfully.

On both occasions, the patient was not willing to be subjected to any investigations apart from a basic presurgical profile, and hence, serum levels of mercury could not be determined.

These two patients happened *(the authors have used past tense because one of them viz. Case 1 is dead—he went home against medical advice and is known to have died in another hospital due to unknown causes. He was himself an assistant to a Unani physician and was repeatedly taking injections of multiple unknown drugs)* to be close friends and apparently had taken these injections on the advice of patient no: 1 (in case report 1) as an aphrodisiac. Patient no: 2, being less adventurous, stopped after one injection and turned up at the hospital for treatment.

## DISCUSSION

Intravenous injection of elemental mercury is comparatively less harmful as compared to that of its salts or the inhalation of mercury vapour. The heavy metal tends to gravitate to dependent areas and extravasates into tissues. When carried by circulation, it tends to lodge in the lungs and mediastinum and is also known to reach other organs over a period of time. Mercury can be removed from the body by oral treatment with 2,3-Dimercaptopropane-1-sulphonate and *meso* -2,3-Dimercaptosuccinic acid.[1,12,13]

Typical symptoms of acute mercury poisoning via inhalation rather than injection, include those of gastroenteritis, ulceromenbranous colitis and stomatitis.[1] Pleuritic chest pain has been reported whereas symptoms of renal and central nervous involvement were less common.[3] Elemental mercury poisoning has also been implicated in the causation of cortical myoclonus.[20] Abscess formation is the most common local presentation.[19]

As chelation therapy removes 1 mg of mercury per day only, it is best to physically remove as much of mercury as possible from all the accessible areas and use chelation therapy for the remaining mercury. There are several long-term complications of retained mercury which include microscopic inflammation of the lung, axonopathy of various nerves, asthenozoospermia, renal impairment, liver toxicity, and cardiac granuloma.[2,14–17]

In case no.1, it is difficult to come to any conclusion as we do not have information about the duration of exposure and the volume of mercury injected.

In contrast, in case no.2, all the metallic elemental mercury was removed on two occasions and the patient has led an uneventful life showing no clinical signs of mercury poisoning in the follow-up period of two years.

Just incision and drainage is of no use in such cases. Extensive exposure and physical removal of mercury along with surrounding tissue, if necessary, is essential.[18] Although our patient did not allow us to investigate him in detail,[19,20] a clinical examination reveals no cause for suspecting any other organ damage.

## References

[1] Winker R, Schaffer AW, Konnaris C, Barth A, Giovanoli P, Osterode W (2002). Health consequences of an intravenous injection of metallic mercury. Int Arch Occup Environ Health.

[2] McFee RB, Caraccio TR (2001). Intravenous mercury injection and ingestion: Clinical manifestations and management. J Toxicol Clin Toxinol.

[3] Giombetti RJ, Rosen DH, Kuezmierczyk AR, Marsh DO (1988). Repeated suicide attempts by the intravenous injection of elemental mercury. Int J Psychiatry Med.

[4] Oliver RM, Thomas MR, Cornaby AJ, Neville E (1987). Mercury pulmonary emboli following intravenous self-injection. Br J Dis Chest.

[5] Hannigan BG (1978). Self-administration of metallic mercury by intravenous injection. Br Med J.

[6] Kumar A, Jain R, Sawhney S, Geol AK, Chattopadhyay K (1992). Intravenous administration of metallic mercury with homicidal intent. J Assoc Physicians India.

[7] Givica-perez A, Santana-Montesdeoca JM, Diaz-Sanchez M, Martinez-Lagares FJ, Castaneda WR (2001). Deliberate, repeated self-administration of metallic mercury injection: Case report and review of the literature. Eur Radiol.

[8] dell'Omo M, Muzi G, Bernard A, Filiberto S, Lauwerys RR, Abbritti G (1997). Long term pulmonary and systemic toxicity following intravenous mercury injection. Arch Toxicol.

[9] Torres-Alanis O, Garza-Ocanas L, Pineyro-Lopez A (1997). Intravenous self-administration of metallic mercury: Report of a case with a 5-year follow-up. J Toxicol Clin Toxicol.

[10] dell'Omo M, Muzi G, Bernard A, Lauwerys RR, Abbritti G (1996). Long-term toxicity of intravenous mercury injection. Lancet.

[11] Ambre JJ, Welsh MJ, Svare CW (1977). Intravenous elemental mercury injection:blood levels and excretion of mercury. Ann Intern Med.

[12] Buchet JP, Lauwerys RR (1989). Influence of 2,3-dimercaptopropone-1-sulfonate and dimercaptosuccinic acid on the mobilization of mercury from tissues of rats pretreated with mercuric chloride, phenylmercury acetate or mercury vapors. Toxicology.

[13] Eyer F, Felgenhauer N, Pfab R, Drasch G, Zilker T (2006). Neither DMPS nor DMSA is effective in quantitative elimination of elemental mercury after intentional IV injection. Clin Toxicol (Phila).

[14] Murray KM, Hedgepeth JC (1988). Intravenous self-administration of elemental mercury: Efficacy of dimercaprol therapy. Drug Intell Clin Pharm.

[15] Ambre J (1977). Consequences of intravenous mercury injections. N Engl J Med.

[16] Rodrigues IM, Hopkinson ND, Harris RI (1986). Pulmonary embolism associated with self-administration of mercury. Hum Toxicol.

[17] Kedziora A, Duflou J (1995). Attempted suicide by intravenous injection of mercury: A rare cause of cardiac granulomas, A case report. Am J Forensic Med Pathol.

[18] Netscher DT, Friedland JA, Guzewicz RM (1991). Mercury poisoning from intravenous injection: Treatment by granuloma resection. Ann Plast Surg.

[19] Spier W, Schulte J Local Therapy in a case of intravenous mercury injection.

[20] Ragothaman M, Kulkarni G, Ashraf VV, Pal PK, Chickabasavaiah Y, Shankar SK (2007). Elemental mercury poisoning probably causes cortical myoclonus. Movement Disorders.

